# Fluoroquinolone-derived fluorescent probes for studies of bacterial penetration and efflux†
†Electronic supplementary information (ESI) available: LCMS analyses of probes **3** and **4**. See DOI: 10.1039/c9md00124g


**DOI:** 10.1039/c9md00124g

**Published:** 2019-05-17

**Authors:** M. Rhia L. Stone, Muriel Masi, Wanida Phetsang, Jean-Marie Pagès, Matthew A. Cooper, Mark A. T. Blaskovich

**Affiliations:** a Institute for Molecular Bioscience , The University of Queensland , Brisbane , QLD 4072 , Australia . Email: m.blaskovich@uq.edu.au; b Membranes et Cibles Thérapeutiques , UMR_MD1 , Inserm U1261 , Aix-Marseille Univ & IRBA , Facultés de Médecine et de Pharmacie , 27 Bd Jean Moulin , 13005 Marseille , France

## Abstract

Fluorescent probes derived from the fluoroquinolone antibiotic ciprofloxacin were synthesised using a Cu(i)-catalysed azide–alkyne cycloaddition (CuAAC) to link a ciprofloxacin azide derivative with alkyne-substituted green and blue fluorophores. The azide (**2**) and fluorophore (**3** and **4**) derivatives retained antimicrobial activity against Gram-positive and Gram-negative bacteria. The use of confocal fluorescent microscopy showed intracellular penetration, which was substantially enhanced in the presence of carbonyl cyanide 3-chlorophenylhydrazone as an efflux pump inhibitor in *Escherichia coli*.

## Abstract 2

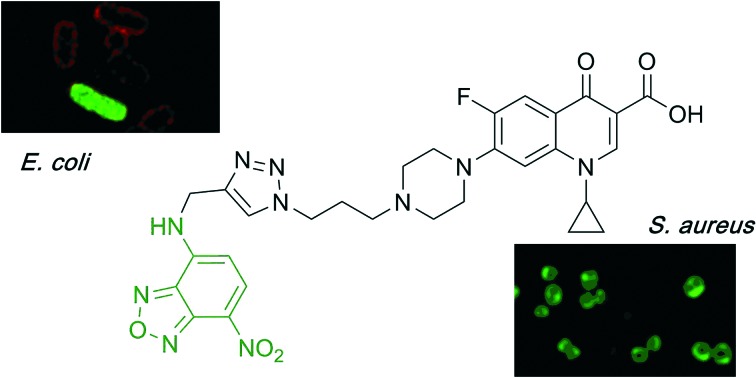
Fluorescent probes derived from the fluoroquinolone antibiotic ciprofloxacin were synthesised using a Cu(i)-catalysed azide–alkyne cycloaddition (CuAAC) to link a ciprofloxacin azide derivative with alkyne-substituted green and blue fluorophores.

## Introduction

Bacterial infections are an increasing global concern, with rising rates of antimicrobial resistance coupled with a near-empty antibiotic pipeline^1^ leading to agencies such as the United Nations, the World Health Organisation, the United States Centre for Disease Control and Prevention and the Wellcome Trust all urging for action to combat this threat. There is now significant financial encouragement to advance both new antibiotics and novel non-antibiotic approaches to treat multidrug-resistant bacteria through initiatives such as CARB-X (Combating Antibiotic-Resistant Bacteria Biopharmaceutical Accelerator) and the Novo REPAIR Impact Fund. However, these efforts must be supported by fundamental studies that examine the underpinning chemical biology of antibiotic translocation across bacterial envelopes, especially that of Gram-negative bacteria, and key aspects of bacterial growth, division, metabolism and resistance.

Fluorescent probes are versatile reporters of biological activity in cells. There are literally thousands of probes reported for use with mammalian cells, but there is a comparative paucity of agents suitable for the study of microbial processes. During the past few years, fluorescent-based approaches have been developed to determine intracellular concentrations of antibiotics in bacterial populations of *Enterobacteriaceae* by using fluoroquinolones, a class of antibiotics with intrinsic fluorescence properties. More recently, this approach has been extended to the use of deep-UV synchrotron radiation for direct observation of the intracellular antibiotics in single bacterial cells.^2^–^7^ However, limitations include (i) the high facility cost and low output of these techniques for single cell analysis; and (ii) the limited availability of classes of intrinsically fluorescent antibiotics. In order to continue these investigations, we have been developing a toolset of fluorescent probes derived from major classes of antibiotics, developing probes that retain the properties of the parent antibiotic, but have high fluorescence. Chemical probes, particularly antibiotics,^8^ can be applied to unravel complex biological pathways and validate new biological targets.^9^,^10^ There have only been a limited number of antibiotic-based fluorescent probes reported to date,^11^ but they have been applied to a range of useful studies including antibiotic localisation, and mode of action studies,^12^–^19^ biological target identification and validation,^20^–^22^ and screening assays.^23^ Somewhat surprisingly, a substantial number of these probes were not assessed to ensure they retain antimicrobial activity, raising concerns over how accurately they reflect the properties of the parent antibiotic.

To date we have reported on probes based on oxazolidinone (linezolid, protein synthesis inhibitor *via* binding to 50S ribosomal subunit)^24^ and trimethoprim (dihydrofolate reductase inhibitor)^25^ antibiotics. Our strategy employs addition of an azide ‘handle’ to the core antibiotic at a position known to be tolerant of substitution, with the goal of retaining antimicrobial activity in the final probes. The Cu-catalyzed azide–alkyne cycloaddition (CuAAC) reaction is then used to append alkyne-functionalised fluorophores *via* a stable and biocompatible triazole ring linker. This approach allows for the facile introduction of multiple colour fluorophores to a common antibiotic template. For antibiotics hitting intracellular targets, it is particularly important to use smaller fluorophores to maximise the penetrance of the compound into the cytosol, particularly with Gram-negative bacteria. For this reason, we have selected the green nitrobenzofurazan (NBD, *M* = 164 g mol^–1^) and blue 7-(dimethylamino)-coumarin-4-acetic acid (DMACA, *M* = 261 g mol^–1^) fluorophores, as they are relatively small compared to more common fluorophores such as Oregon Green (*M* = 412 g mol^–1^) or rhodamine B (*M* = 479 g mol^–1^). They are also readily modified with an alkyne substituent.^24^

Here, we describe the preparation and characterization of fluorescent probes derived from the fluoroquinolone antibiotic ciprofloxacin. Ciprofloxacin is a second-generation fluoroquinolone antibiotic that is active against a broad spectrum of Gram-positive and -negative bacteria. It acts in a bactericidal manner, inhibiting DNA gyrase and topoisomerase IV, thereby impeding DNA synthesis. The AcrAB-TolC multidrug efflux pump plays a major role in controlling the intracellular level of fluoroquinolones in *Escherichia coli* and closely related *Enterobacteriaceae* such as *Enterobacter aerogenes*, with an average of 3–4 fold difference in antibiotic activity with pump functionality.^4^,^6^,^7^ Consequently, the activity of the pump also dictates drug susceptibility in these species. In *Pseudomonas aeruginosa*, the MexAB-OprM complex is the major multidrug efflux system contributing to intrinsic multidrug resistance. It is a tripartite complex homologous to the *E. coli* AcrAB-TolC efflux pump, and is associated with fluoroquinolone resistance. Fluorophore-coupled ciprofloxacin derivatives with similar accumulation properties as the parent antibiotics would allow the opportunity to evaluate the efficacy of efflux pump inhibitors (EPIs) that could be used as therapeutic adjuvants.^26^

## Results and discussion

**Scheme 1:**
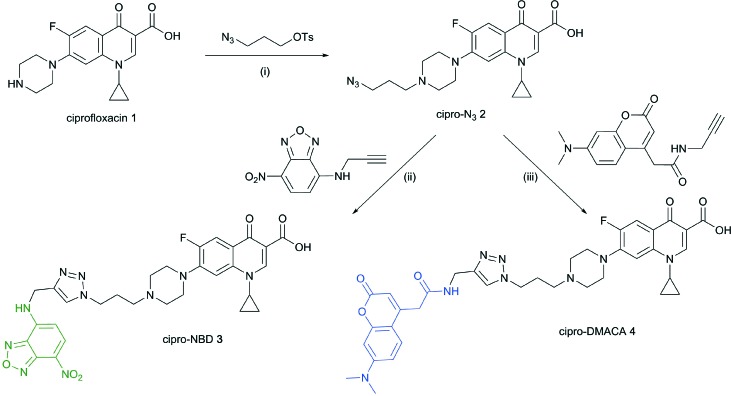
Reagents and conditions: (i) NaHCO_3_, NaI, acetonitrile, reflux 12–24 h, quantitative; (ii) CuSO_4_, sodium ascorbate, DMF/H_2_O, 57%; (iii) CuSO_4_, sodium ascorbate, DMF/H_2_O, 12%.

Cipro-azide **2** was prepared from ciprofloxacin **1***via* alkylation with tosylated 3-azidopropanol in quantitative yield (Scheme 1). Azide **2** was then subjected to CuAAC using copper(ii) sulfate and sodium ascorbate as a reducing agent, coupling with NBD- and DMACA-alkynes in moderate yields.

Antibacterial activity of all compounds was first tested against representative species of drug-susceptible American Type Culture Collection (ATCC) bacterial strains, including both Gram-positives (*Staphylococcus aureus* ATCC25923, *Bacillus subtilis* ATCC6051 and *Enterococcus faecium* ATCC35667) and Gram-negatives (*Klebsiella pneumoniae* ATCC13883, *Acinetobacter baumannii* ATCC19606, *P. aeruginosa* ATCC ATCC27853 and *E. coli* ATCC25922). Standard broth microdilution assays were used for determination of minimal inhibitory concentrations (MICs, see Experimental section). Cipro-azide **2** maintained excellent to good antibacterial activity against both Gram-positive and -negative susceptible bacterial species (Table 1). Upon addition of the fluorophore moieties, MICs generally increased across the board, though with moderate to good activity observed.

**Table 1:** MIC (minimum inhibitory concentrations) of fluoroquinolone derivatives

Species	Strain	MIC (μg mL^–1^)			
		Ciprofloxacin 1	Cipro-N_3_ 2	Cipro-NBD 3	Cipro-DMACA 4
*Staphylococcus aureus*	ATCC 25923	0.125–0.5	0.25	32–≥64	16
*Bacillus subtillis*	ATCC 6051	0.313–0.06	0.06–0.25	8	4
*Enterococcus faecium*	ATCC 35667	1–8	16–≥64	32	32–≥64
*Klebsiella pneumoniae*	ATCC 13883	0.015–0.06	0.125–0.25	8–16	4–32
*Acinetobacter baumannii*	ATCC 19606	0.5–1	0.25–0.5	32–≥64	32
*Pseudomonas aeruginosa*	ATCC 27853	0.25–1	8–≥64	32–≥64	32–≥64
	PAO750 PAO397 Δ^*a*^	≤0.25	32–≥64	4	2–8
*Escherichia coli*	ATCC 25922	≤0.004	0.06–0.25	8	2
	MB4902 Δ*lpxC*	≤0.004	0.25–1	1–4	2
	MB5747 Δ*tolC*	≤0.004	0.25–1	0.125–0.25	0.5–1
	MB5746 Δ*lpxC*, Δ*tolC*	≤0.004	0.25–1	0.06–0.125	0.5–1

^*a*^Delta efflux pump mutant: Δ*mexAB-oprM*, Δ*mexCD-oprJ*, Δ*mexEF-oprN*, Δ*mexJKL*, Δ*mexXY*, Δ*opmH*, Δ*pscC*.

It is well known that the susceptibility of Gram-negative bacteria to antibiotics is defined by two opposing fluxes across the two membranes of these species.^27^ First, influx across the outer membrane is significantly slowed due to the presence of lipopolysaccharides but occurs through the narrow channel of porins.^28^,^29^ Second, tripartite multidrug efflux pumps mediate active efflux across the two membranes towards the extracellular medium.^30^ Consequently, the observed reduced activity of the modified ciprofloxacin derivatives could be due to decreased influx and/or increased efflux. Therefore, MICs were determined in efflux-deficient derivatives of *P. aeruginosa* (a PAO1 derivative mutant with 6 major tripartite efflux pumps deleted^31^) and *E. coli* (Δ*tolC*)^32^ to assess the role of efflux, and in an outer membrane compromised mutant of *E. coli* (Δ*lpxC*)^32^ to assess the role of influx.

The cipro-N_3_ intermediate **2** generally retained similar activity to the parent antibiotic, as might be expected for the relatively small chemical change. In contrast, the cipro-DMACA probe **4** was uniformly around 50-fold less active than ciprofloxacin against a range of Gram-positive and Gram-negative strains. Surprisingly, neither the Δ*lpxC* nor the Δ*tolC* mutants resulted in significantly improved activity. In contrast, in the *P. aeruginosa* multiple efflux pump knockout, substantial improvement was seen compared to an ATCC strain. The cipro-NBD probe **3** had a generally similar profile to the cipro-DMACA **4** probe against wild-type strains, though with some strain dependent variations (*e.g.* less active *vs. S. aureus*, more active *vs. E. faecium*). The greatest variation was against the *E. coli* mutants, where enhanced activity (up to 100-fold) was seen against both efflux pump and membrane mutants.

**Fig. 1:**
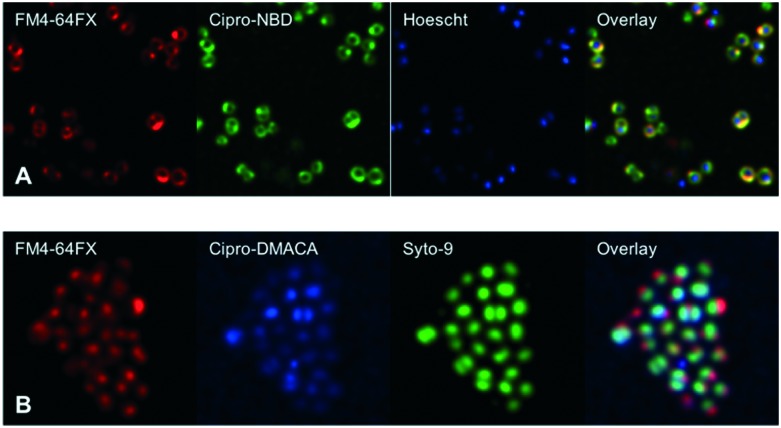
Confocal fluorescent microscopy of live *S. aureus* labelled with A: red FM4-64FX membrane dye, green cipro-NBD **3**, blue nucleic acid dye Hoescht 33342, and overlay; B: red FM4-64FX membrane dye, blue cipro-DMACA **4**, green nucleic acid dye Syto-9, and overlay.

**Fig. 2:**
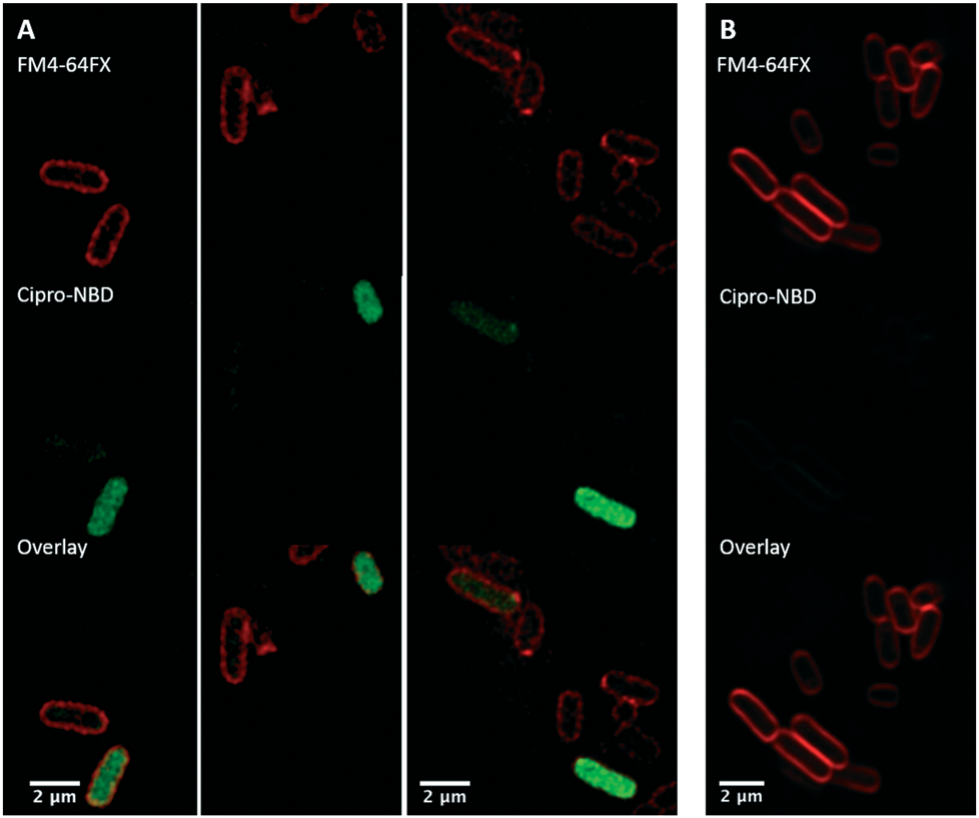
Confocal fluorescent microscopy of: live *E. coli* AG102 labelled with: red FM4-64FX membrane dye and green cipro-NBD probe **3**, with (A, several fields of view showing internalisation) and without (B) efflux pump inhibitor, showing no internalisation.

Next, fluorescent probes were tested for labelling intact bacteria in confocal microscopy, using *S. aureus* and *E. coli* as model organisms. *E. coli* AG102 is a *mar* mutant derivative of the wild type K12 AG100 that overexpresses the major AcrAB multidrug efflux pump.^33^ Previous studies have shown that incubating this strain in the absence or in the presence of the efflux pump inhibitor carbonyl cyanide 3-chlorophenylhydrazone (CCCP) results in a 8–16 fold increase of ciprofloxacin activity, which also correlated with a 3–4 fold increase of the intracellular drug content.^2^,^4^ When AG102 cells were incubated in the presence of the cipro-NBD **3**, very little labelling of the bacteria was observed, due to active removal of the probe by the AcrAB pump (Fig. 2, B). With addition of the efflux pump of CCCP, which collapses the proton-motive force necessary to the pump activity, cipro-NBD **3** accumulation was observed (Fig. 2, A). Interestingly, the double cell labelling in the presence of the antibiotic probe and a specific membrane probe (FM4-64FX) shows that the fluorescence of cipro-NBD **3** correctly localized in the cytoplasm of *E. coli*. However, in the case of the Gram-positive bacteria *S. aureus* with cipro-NBD **3**, accumulation was mostly seen at the membrane (Fig. 1, A), potentially due to the enhanced fluorescence of the NBD fluorophore in lipid environments.^34^ Using the cipro-DMACA probe **4**, internal localisation was observed for both Gram-positive (Fig. 1, B) and Gram-negative bacteria (images not shown). The increased internal localisation seen in *S. aureus* for **4** compared to **3** may reflect the slightly better antibacterial activity of **4**, coupled with differences in the physicochemical character of the probes affecting localization.

## Experimental

### Materials and methods

All materials, unless otherwise noted, were obtained from commercial suppliers and used without further purification. Non-aqueous reactions were conducted under an inert atmosphere of nitrogen. Reactions were monitored by thin layer chromatography (TLC) or analytical liquid chromatography mass spectrometry (LCMS). Analytical TLC was performed on Merck TLC alumina sheets pre-coated with Silica Gel 60 F254, and compounds were visualized using UV lamp and appropriate TLC stains. Analytic LCMS was performed on either Shimadzu LCMS-2020 or Agilent 1200 series LCMS using 0.05% formic acid in water as solvent A and 0.05% formic acid in acetonitrile as solvent B. A Grace Reveleris chromatography system was used for compound purification. ^1^H (600 MHz) and ^13^C (125 MHz) NMR spectra were obtained using a Bruker Avance-600 spectrometer equipped with a TXI cryoprobe. Chemical shifts are reported relative to the residual solvent signals in parts per million (*δ*) (DMSO-d_6_: ^1^H: *δ* 2.50, ^13^C: *δ* 39.5). High resolution mass spectrometry (HRMS) was performed on a Bruker Micro TOF mass spectrometer using (+)-ESI calibrated to NH_4_OAc. Microscopy was carried out on a Leica STED 3X Super Resolution Microscope with White Light Laser excitation. High performance glass cover slips by Zeiss (18 × 18 mm) and superfrost glass microscope slides by Menzel (26 × 76 mm), and Vectashield or Cygel mounting media were used.

### Synthetic procedures

#### Synthesis of compound **2**, Cipro-N_3_

Azide-functionalised ciprofloxacin 2 was prepared following a modified version of the literature procedure,^35^ with a mixture of ciprofloxacin (40 mg, 0.12 mmol), tosylated 3-azidopropanol^24^ (154 mg, 0.6 mmol), NaI (16 mg) and powdered NaHCO_3_ (10 mg. 0.12 mmol) in acetonitrile (15 mL) refluxed for 12–24 h. The reaction was monitored by TLC (MeOH : CH_2_Cl_2_, 1 : 9). When complete the mixture was filtered, washed with excess MeOH : CH_2_Cl_2_ (1 : 1), and the combined filtrates evaporated to dryness. Chromatographic purification by MPLC (0–100% ACN (0.05% FA) in H_2_O (0.05% FA)) produced 60 mg of white solid (quantitative yield). ^1^H NMR (600 MHz, DMSO-*d*_6_) *δ* 9.82 (m, 1H), 8.70 (s, 1H), 7.98 (d, *J* = 12.8 Hz, 1H), 7.61 (d, *J* = 7.2 Hz, 1H), 6.51 (s, 1H), 3.91 (br s, 1H), 3.85 (tt, *J* = 7.5, 4.1 Hz, 2H), 3.68 (br s, 2H), 3.50 (m, 4H), 3.34 (m), 1.96 (br s, 2H), 1.31 (d, *J* = 6.9 Hz, 2H), 1.20 (br s, 2H).

#### Synthesis of compound **3**, cipro-NBD

Following methodology described by Dixit *et al.*,^36^ cipro-N_3_**2** (19.6 mg, 0.0470 mmol) was dissolved in 1 mL water and 1 mL DMF, then NBD alkyne^24^ (11.2 mg, 0.0513 mmol) was added. Ascorbic acid (50 μL, 400 mM in water, 0.0196 mmol) was added, followed by copper sulfate (20 μL, 450 mM in water, 0.00893 mmol). The reaction was heated to 60 °C for 1 hour, then cooled. The reaction was direct injected onto MPLC (0–100% ACN (0.05% FA) in H_2_O (0.05% FA)) to give pure **2** as an orange solid (17.1 mg, 57%). LCMS: *R*_t_ = 5.60 min, >90% purity, [M + H]^+^ = 633.5; ^1^H NMR (600 MHz, DMSO-*d*_6_) *δ* 9.90 (t, *J* = 6.2 Hz, 1H), 8.68 (s, 1H), 8.53 (d, *J* = 8.8 Hz, 1H), 8.17 (s, 1H), 7.96 (d, *J* = 12.7 Hz, 1H), 7.60 (d, *J* = 7.3 Hz, 1H), 6.51 (d, *J* = 8.9 Hz, 1H), 4.78 (s, 2H), 4.47 (t, *J* = 6.9 Hz, 2H), 3.84 (dt, *J* = 7.2, 3.3 Hz, 2H), 3.49 (m, 1H), 3.41 (t, *J* = 5.3 Hz, 1H), 3.34–3.26 (m, 3H), 3.21 (t, *J* = 8.1 Hz, 2H), 2.89 (s, 2H), 2.73 (s, 1H), 2.33–2.26 (m, 2H), 1.31 (m, 2H), 1.23 (s, 3H), 1.19 (m, 2H).

#### Synthesis of compound **4**, cipro-DMACA

Following methodology described by Dixit *et al.*,^36^ cipro-N_3_**2** (19.5 mg, 0.0468 mmol) was dissolved in 1 mL water and 1 mL DMF, then DMACA alkyne^24^ (11.9 mg, 0.0419 mmol) was added. Ascorbic acid (50 μL, 400 mM in water, 0.0203 mmol) was added, followed by copper sulfate (25 μL, 450 mM in water, 0.00953 mmol). The reaction was heated to 60 °C for 1 hour, then cooled. The reaction was direct injected onto MPLC (0–100% ACN (0.05% FA) in H_2_O (0.05% FA)) to give pure **47** as a yellow solid (3.9 mg, 12%). LCMS: *R*_t_ = 3.27 min, >90% purity, [M + H]^+^ = 699.2; ^1^H NMR (600 MHz, DMSO-d_6_) *δ* 8.74 (t, *J* = 6.5 Hz, 1H), 8.67 (s, 1H), 8.24 (d, *J* = 8.7 Hz, 1H), 7.92 (m, 2H), 7.67 (d, *J* = 9.3 Hz, 1H), 7.56 (d, *J* = 7.3 Hz, 1H), 7.51 (d, *J* = 8.9 Hz, 1H), 6.68 (dd, 1H), 5.98 (s, 1H), 4.55 (s, 1H), 4.41 (s, 2H), 4.32 (d, *J* = 5.6 Hz, 2H), 3.83 (dd, *J* = 8.0, 3.9 Hz, 2H), 3.63 (s, 3H), 3.49 (m 2H), 3.41 (d, *J* = 5.7 Hz, 2H), 3.01 (s, 6H), 1.23 (s, 12H), 1.14 (m, 6H).

### Microbiology assays

#### Bacteria isolates were obtained from the American Type Culture Collection

(ATCC; Manassas, VA, USA), Merck Sharp & Dohm (Kenilworth, NJ),^32^ Herbert Schweizer at Colorado State University^31^ and independent clinical isolate collections. Bacteria were cultured in cation-adjusted Muller Hinton broth (CAMHB) (Bacto laboratories, Cat. no. 211443) at 37 °C overnight. A sample of each culture was then diluted 50-fold in CAMHB and incubated at 37 °C for 1.5–3 h. The compounds were serially diluted two-fold across the wells, with concentrations ranging from 0.0078 μg mL^–1^ to 128 μg mL^–1^, plated in duplicate. The resultant mid-log phase cultures were diluted to the final concentration of 5 × 10^5^ CFU mL^–1^, then 50 μL was added to each well of the compound-containing 96-well plates (Corning; Cat. No 3641, NBS plates), giving a final compound concentration range of 0.0039 μg mL^–1^ to 64 μg mL^–1^. All the plates were covered and incubated at 37 °C for 18–24 h with the MIC defined as the lowest compound concentration at which no bacterial growth was visible (*n* ≥ 3).

### Fluorescence microscopy

Glycerol stocks of bacterial strains were streaked on LB agar and grown overnight at 37 °C. Single colonies were then picked and cultured overnight in CAMHB at 37 °C, then diluted ∼40-fold and grown to OD_600_ = 0.4–0.6. Cultures were centrifuged at 14 000 rpm and broth decanted. The pellets were suspended in HBSS, centrifuged, and the liquid decanted. The pellets were re-suspended in 200–500 μL HBSS spiked with the appropriate probe (50–100 μM) and CCCP (10 μM), then incubated at 37 °C for 30–45 min. The cultures were spun down, decanted, and washed with HBSS, then nucleic acid staining was carried out using Hoescht 33 342 (5 μg mL^–1^) or Syto-9 (10 μM) for 20 min. Following washing, membrane labelling was carried out using FM4-64FX (5 μg mL^–1^) for 5 min on ice. After centrifuging and decanting, the final pellet was washed then resuspended in 50 μL of HBSS. 2 μL of this suspension was spread onto a cover slip then dried, then mounted onto a microscope slide using Vectashield mounting medium (13 μL), and the edges sealed using clear nail polish. Alternatively, the washed pellets were suspended in 15 μL Cygel and mounted onto slides.

## Conclusions

We have prepared fluorescent derivatives of the fluoroquinolone antibiotic ciprofloxacin as a potentially more sensitive tool to track antibiotic penetration and accumulation. Our preliminary studies have shown that the probes could readily be used to study efflux and drug accumulation of the fluoroquinolones, though microscopy indicates considerable heterogeneity between individual cells. The increased size of the probes and characteristics of the fluorophore do result in modified activity and variations in sensitivity (compared to the parent antibiotic) depending on bacterial strain, so they must be applied judiciously following determination of the MIC value against the bacterial strain of interest. Future experiments will explore in greater detail probe localisation, heterogeneity in uptake seen between cells, the synergy of efflux with membrane permeability, and changes in uptake following other types of efflux pump inhibition, such as with an acrAB-defective strain of *E. coli* (*i.e.* AG100A). In addition to their utility in studies investigating antibiotic localisation, bacterial penetration and mode of action, these probes could find utility in screening assays to find and evaluate efflux pump inhibitors (EPIs) that could be used as therapeutic adjuvant to overcome fluoroquinolone resistance.

## Conflicts of interest

There are no conflicts to declare.

## Supplementary Material

Supplementary information
