# Supplementary material for: Fluoroquinolone-derived fluorescent probes for studies of bacterial penetration and efflux
Source: Medchemcomm. 2019 May 17;10(6):901–6. doi: 10.1039/c9md00124g (PMC6596217; doi:10.1039/c9md00124g)

Electronic Supplementary Material (ESI) for MedChemComm.
This journal is © The Royal Society of Chemistry 2019

Fluoroquinolone-Derived Fluorescent Probes for Studies of Bacterial Penetration and Efflux

M. Rhia L. Stone,[a] Muriel Masi,[b,d] Wanida Phetsang,[a] Jean-Marie Pagès,[b] Matthew A Cooper[a] and Mark A. T. Blaskovich*[a,c]

Supplementary Material

LCMS Data for cipro-NBD 3

Obtained on a Shimadzu LCMS-2020 LCMS using 0.05% formic acid in water as solvent A and 0.05% formic acid in acetonitrile as solvent B. Zorbax Eclipse XDB-Phenyl column, 3 x 100 mm, 3.5 µm. Method: 5% B for 3 mins, up to 100% B over 8 mins, 5% for 2 mins.

Sample File C:\LabSolutions\Data\RES_2019_04_12\mrls-cipro-nbd_003.lcd
Date Acquired : 12/04/2019 4:58:17 PM Sample Name : mrls-cipro-nbd
Method File : C3_T13_G0009.lcm

Date Acquired	: 12/04/2019 4:58:17 PM	Sample Name	: mrls-cipro-nbd
Sample ID	:	Tray#	: 1
Vial#	: 24	Injection Volume	: 10
Data File	: mrls-cipro-nbd_003.lcd	Method File	: C3_T13_G0009.lcm
Date Processed	: 12/04/2019 5:11:21 PM		

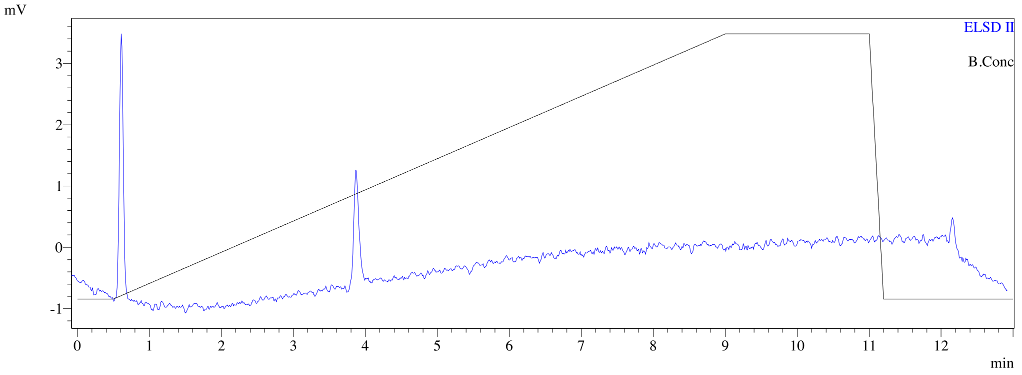

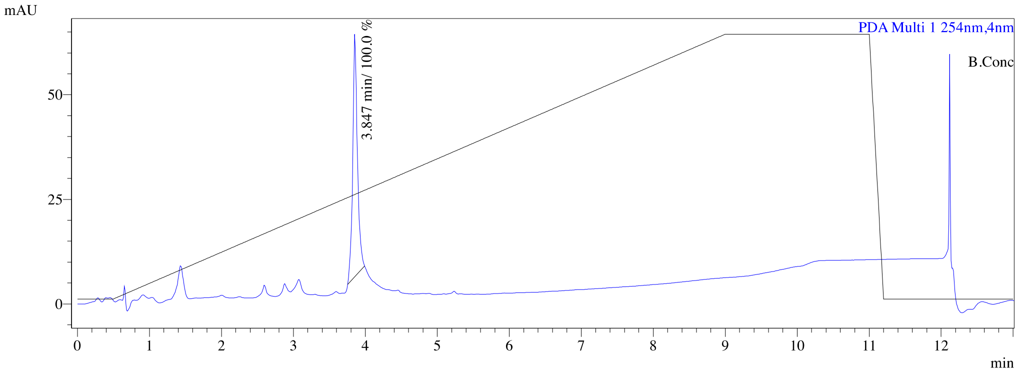

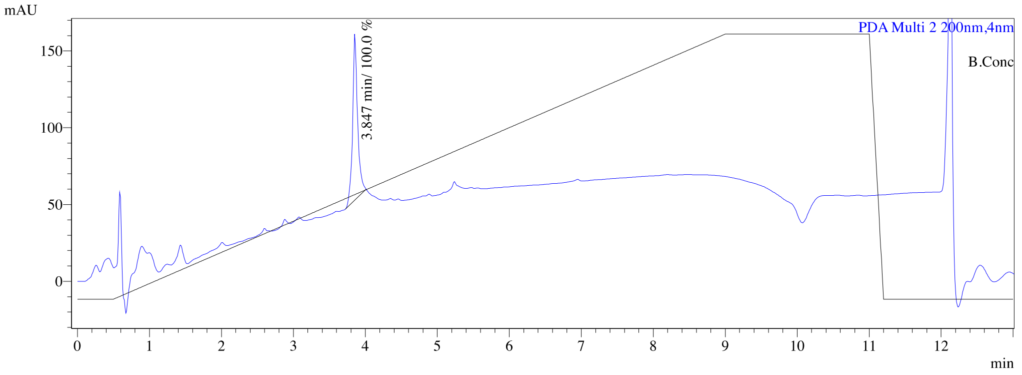

Cooper Group
Institute of Molecular Bioscience
The University of Queenland

Sample File C:\LabSolutions\Data\RES_2019_04_12\mrls-cipro-nbd_003.lcd
Date Acquired : 12/04/2019 4:58:17 PM Sample Name : mrls-cipro-nbd
Method File : C3_T13_G0009.lcm

MS Chromatogram

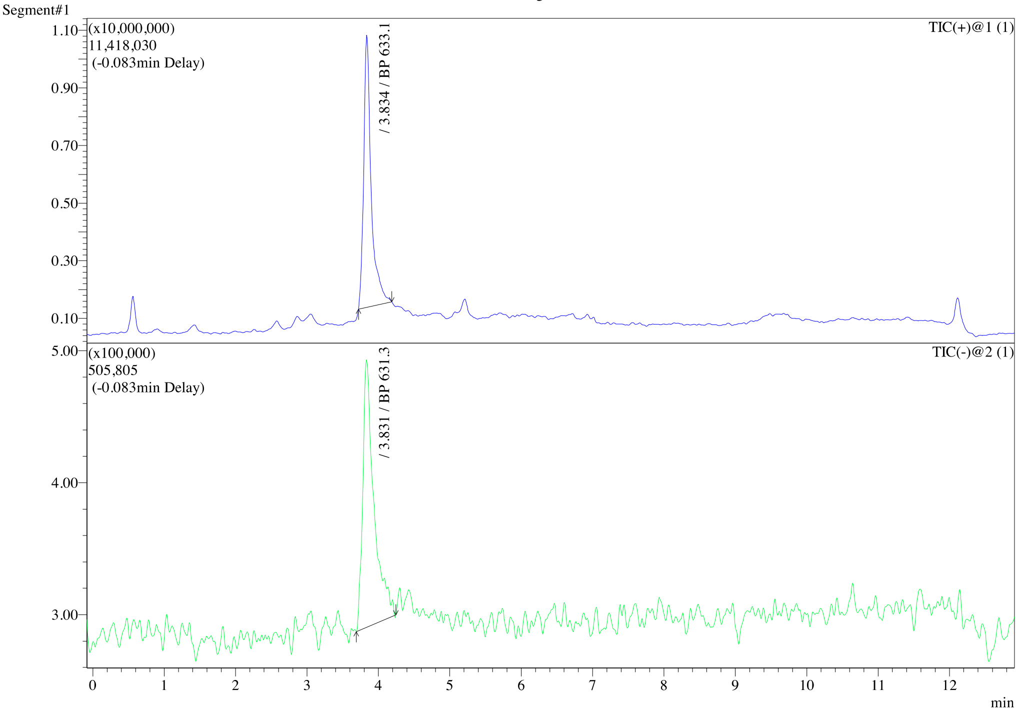

Peak#:1 R.Time:3.831(Scan#:1342)
Spectrum Mode:Averaged 3.693-4.247(1296-1486)
BG Mode:Averaged 4.247-4.253(1486-1488) Segment 1 - Event 2

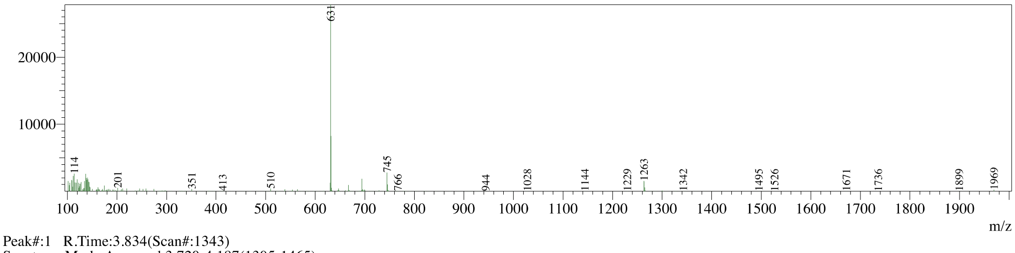

Peak#:1 R.Time:3.834(Scan#:1343)
Spectrum Mode:Averaged 3.720-4.187(1305-1465)
BG Mode:Averaged 4.187-4.193(1465-1467) Segment 1 - Event 1

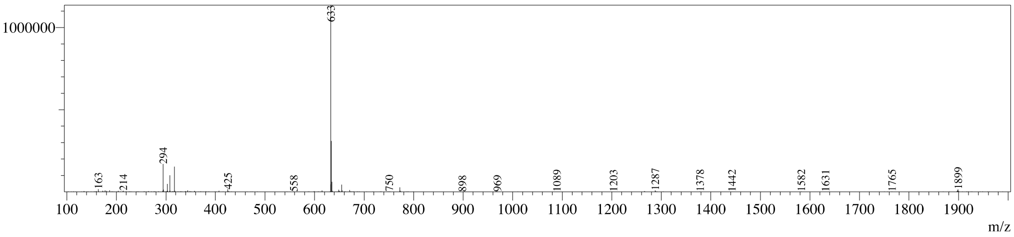

Cooper Group
Institute of Molecular Bioscience
The University of Queensland

Sample File C:\LabSolutions\Data\RES_2019_04_12\mrls-cipro-nbd_003.lcd
Date Acquired : 12/04/2019 4:58:17 PM Sample Name : mrls-cipro-nbd
Method File : C3_T13_G0009.lcm

UV Peak Table mrls-cipro-nbd_003.lcd

ELSD II

Peak#	Ret. Time	Area	Area%	Height	Height%	S/N	Peak Purity Index	Name
Total								

PDA Ch1 254nm

Peak#	Ret. Time	Area	Area%	Height	Height%	S/N	Peak Purity Index	Name
1	3.847	262717	100.000	58096	100.000	17.44	1.000000	
Total		262717	100.000	58096	100.000			

PDA Ch2 200nm

Peak#	Ret. Time	Area	Area%	Height	Height%	S/N	Peak Purity Index	Name
1	3.847	520164	100.000	108332	100.000	3.04	1.000000	
Total		520164	100.000	108332	100.000			

MASS Peak Table ALL MC

Peak#	Ret. Time	Base Peak m/z	Area	Area%	Height	Event#	Compound Name
1	3.834	633.05	69156097	100.000	9452115	1-1	
2	3.831	631.30	2215298	100.000	202817	1-2	
Total			71371395	200.000			

Method Information

<<Pump>>
Mode : Binary gradient
Pump A : LC-20AD
Pump B : LC-20AD
Total Flow : 1.0000 mL/min
B Conc. : 5.0 %
B Curve : 0
PressMax : 3000 psi
PressMin : 200 psi
Solenoid Valve A Name : FCV-11AL
Solenoid Valve A : A-A-A

<<LC Time Program>>

Time	Module	Command	Value	Comment
0.50	Pumps	Pump B Conc.	5	
9.00	Pumps	Pump B Conc.	100	Zorbax Eclipse XDB-Phenyl, 3.0 ×100mm, 3.5 um
11.00	Pumps	Pump B Conc.	100	
11.20	Pumps	Pump B Conc.	5	
13.00	Controller	Stop		

<<Oven>>
Oven Model : CTO-20AC
Enable Oven : Use
Oven Temperature : 40 C
Maximum Temperature : 90 C
Rotary Valve L : FCV-14AH
Rotary Valve R : FCV-14AH
Valve L : 3
Valve R : 3

Cooper Group
Institute of Molecular Bioscience
The University of Queenland

LCMS Data on cipro-DMACA 4

Obtained on an Agilent 1200 series LCMS using 0.05% formic acid in water as solvent A and 0.05% formic acid in acetonitrile as solvent B. Eclipse Phenyl column. Method: 0.3 min 5% B, 3.2 min 100%, 5.2 min 100%, 5.5 min 5%, 7.00 min 5%.

Data File F:\A_MRLSPHD03042A-G1-I2-F13.D
Sample Name: MRLSPhD03042A-G1-I2-F13

===

Acq. Operator : Cooper Seq. Line : 3
Acq. Instrument : Instrument 1 Location : P1-E-03
Injection Date : 7/24/2018 3:58:27 PM Inj : 1
Inj Volume : 1.0 µl
Different Inj Volume from Sequence ! Actual Inj Volume : 20.0 µl
Acq. Method : D:\SHARE\DATA\RES_2018_07_24\SEQ_2018_07_24_02 2018-07-24 15-39-49\C4T07_G0003_U210P.M
Last changed : 3/21/2018 2:15:34 PM by Cooper
Analysis Method : D:\COOPER\METHODS\YN6C_2018_POS1_G0015_U210_254_COLLECT.M
Last changed : 3/8/2019 1:08:18 PM by Cooper Group
Additional Info : Peak(s) manually integrated

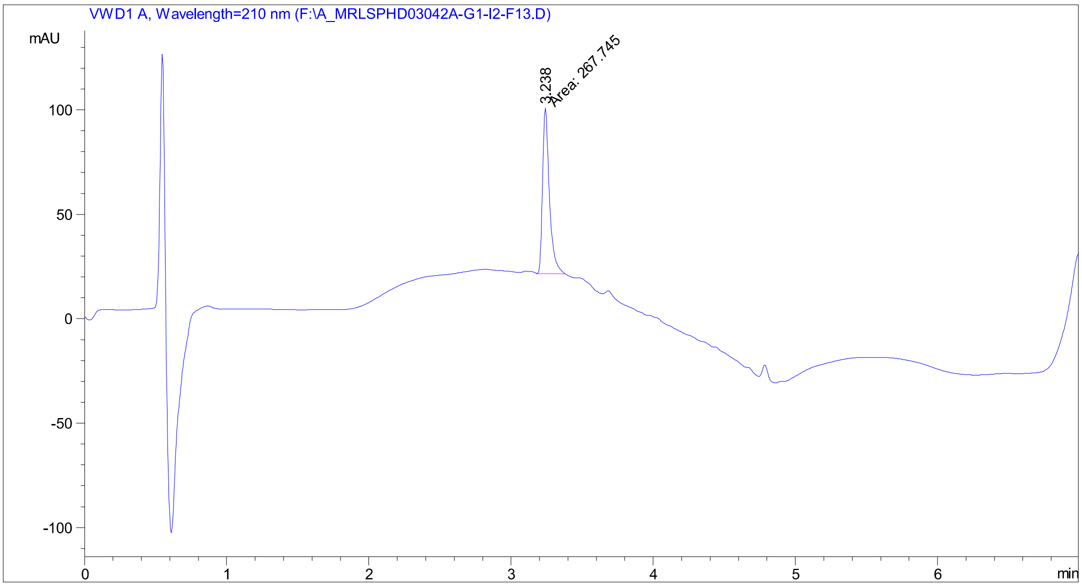

===

Area Percent Report

===

Sorted By : Signal
Multiplier: : 1.0000
Dilution: : 1.0000
Use Multiplier & Dilution Factor with ISTDs

Signal 1: VWD1 A, Wavelength=210 nm

Peak #	RetTime [min]	Type	Width [min]	Area mAU *s	Height [mAU]	Area %
1	3.238	MM	0.0562	267.74527	79.35232	100.0000

Totals : 267.74527 79.35232

===

Sample Name : MRLSPhD03042A-G1-I2-F13
Data File : D:\SHARE\DATA\RES_2018_07_24\SEQ_2018_07_24_02 2018-07-24 15-39-49\A_ ->

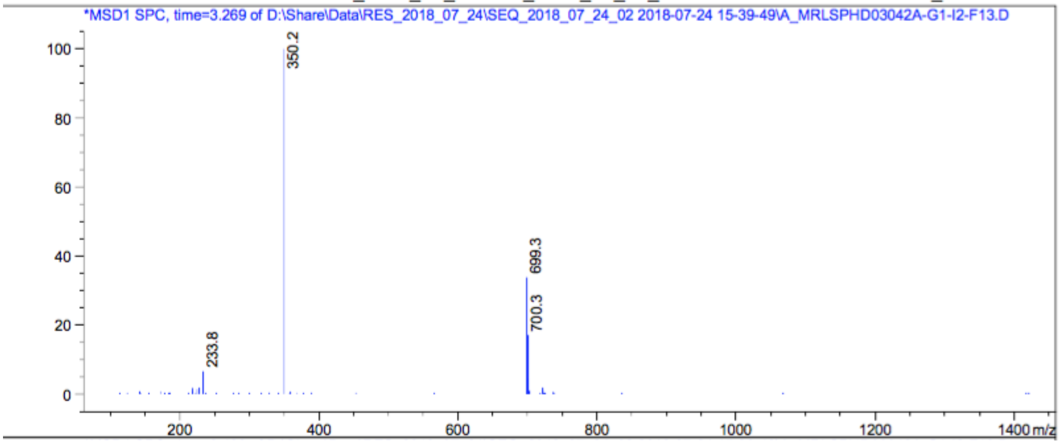

Supplement: Supplementary file 1 [file MD-010-C9MD00124G-s001.pdf]
